# MATH5 controls the acquisition of multiple retinal cell fates

**DOI:** 10.1186/1756-6606-3-36

**Published:** 2010-11-18

**Authors:** Liang Feng, Zheng-hua Xie, Qian Ding, Xiaoling Xie, Richard T Libby, Lin Gan

**Affiliations:** 1 Flaum Eye Institute, University of Rochester School of Medicine and Dentistry, Rochester, NY 14642, USA; 2 Department of Ophthalmology, University of Rochester School of Medicine and Dentistry, Rochester, NY 14642, USA; 3 Department of Neurobiology and Anatomy, University of Rochester School of Medicine and Dentistry, Rochester, NY 14642, USA

## Abstract

*Math5*-null mutation results in the loss of retinal ganglion cells (RGCs) and in a concurrent increase of amacrine and cone cells. However, it remains unclear whether there is a cell fate switch of *Math5*-lineage cells in the absence of *Math5 *and whether MATH5 cell-autonomously regulates the differentiation of the above retinal neurons. Here, we performed a lineage analysis of *Math5*-expressing cells in developing mouse retinas using a conditional GFP reporter (Z/EG) activated by a *Math5*-*Cre *knock-in allele. We show that during normal retinogenesis, *Math5*-lineage cells mostly develop into RGCs, horizontal cells, cone photoreceptors, rod photoreceptors, and amacrine cells. Interestingly, amacrine cells of *Math5*-lineage cells are predominately of GABAergic, cholinergic, and A2 subtypes, indicating that *Math5 *plays a role in amacrine subtype specification. In the absence of *Math5*, more *Math5*-lineage cells undergo cell fate conversion from RGCs to the above retinal cell subtypes, and occasionally to cone-bipolar cells and Müller cells. This change in cell fate choices is accompanied by an up-regulation of NEUROD1, RXRγ and BHLHB5, the transcription factors essential for the differentiation of retinal cells other than RGCs. Additionally, loss of *Math5 *causes the failure of early progenitors to exit cell cycle and leads to a significant increase of *Math5*-lineage cells remaining in cell cycle. Collectively, these data suggest that *Math5 *regulates the generation of multiple retinal cell types via different mechanisms during retinogenesis.

## Introduction

In the developing central nervous system (CNS), different types of neurons are generated from a common pool progenitors in a phylogenetically conserved order. Though it is thought that the sequential generation of CNS neurons is regulated by both extrinsic and intrinsic factors, it is not well understood what the intrinsic factors are and how they determine the neuronal birth order. Vertebrate retinas consist of six major neuronal cell types and one glial cell type that originate from a common pool of retinal progenitors [1,2] and are arranged in three well-defined cellular layers. The primary light-sensing neurons, cones and rods, are located in the outer nuclear layer (ONL). The interneurons, amacrine, bipolar and horizontal cells, and Müller cells compose the inner nuclear layer (INL). The ganglion cell layer (GCL) contains displaced amacrine cells and retinal ganglion cells (RGCs). The generation of these retinal cells follows a defined sequence that ganglion, horizontal, amacrine, and cone cells are the first-born retinal cell types, and rod, bipolar and Müller cells are generated later [3]. Loss-and gain-of-function studies have demonstrated that transcription factors of the basic helix-loop-helix (bHLH) and homeodomain (HD) classes play key roles in retinal cell fate determination. The retinogenic bHLH factors, such as MASH1, MATH3, NGN2, and NEUROD1, are essential for the specification of major retinal cell types via a combined function with HD proteins CHX10, SIX3 and PAX6 [4-8]. Though alterations in the expression of above genes often lead to an increase or decrease in one or more retinal cell types, it is not clear whether the change in cell types arise from cell fate switch due to the lack of cell lineage analysis.

The vertebrate homolog of *Drosophila atonal *(*ato*), *Ath5 *(*atonal *homolog 5), is a key regulator of retinogenesis. Null mutations of *ath5 *lead to agenesis of nearly all RGCs in mice and fish and to a concurrent increase of cone and amacrine cells [9-11]. Previous cell lineage studies using the Cre-loxP recombination system in mice showed that during normal retinal development *Math5*-lineage cells differentiate into ganglion, horizontal, cone, and amacrine cells [12]. Nevertheless, it remains unknown what the cell fate choices of these *Math5*-lineage cells are in *Math5*-null retinas and how MATH5 regulates the differentiation of non-RGCs. Furthermore, the effect of *Math5*-null mutation on retinal progenitors is not fully understood. Here, we demonstrate that loss of *Math5 *leads to an increase of cone, rod, and the displaced amacrine cells originating from *Math5*-lineage cells and infrequently to the ectopic formation of cone-bipolar and Müller glial cells from *Math5*-lineage cells. The observed cell fate conversion is accompanied by the premature expression of non-RGC retinogenetic factors. Without *Math5*, an increased number of *Math5*-lineage cells remain in cell cycle or undergo apoptosis. The number of proliferating progenitors is transiently increased during early retinogenesis and is reduced in the postnatal retina. Thus, our analysis unveils a comprehensive function of *Math5 *in the generation of multiple retinal cell types during the retinal development.

## Results

### Altered cellular composition in *Math5*-null retina

**Figure 1:**
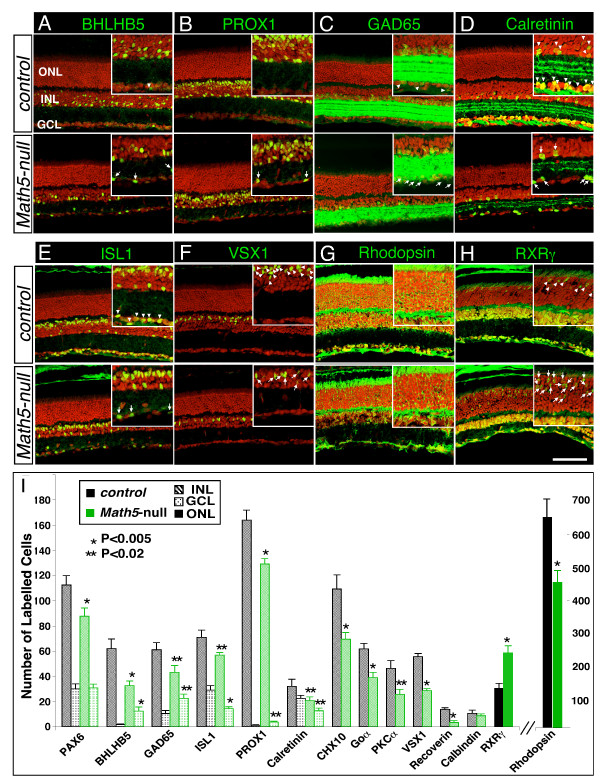
**Effects of *Math5*-null mutation on retinal cell types**. (A-H) Retinal sections from P28 were immunolabeled with cell type-specific markers (green) and nuclear counterstained with propidium iodide (PI, red). Loss of *Math5 *leads to an increase in the displaced amacrine cells immunoreactive for BHLHB5 (A), PROX1 (B) and GAD65 (C) in the GCL, and to a severe loss of calretinin+ (D) and ISL1+ (E) cells in the INL and the GCL. Furthermore, in the ONL, there is a marked reduction in VSX1+ cone bipolar cells (F) and rhodopsin^+ ^rod photoreceptors (G), whereas the RXRγ+ cone photoreceptors are increased (H). (I) Quantification of cells expressing specific markers in the ONL, INL and the GCL per 350 μm length of retinal sections. Eyes from five mice were analyzed per genotype. Arrows in the insets indicate cells expressing specific markers in the wild type retina, while arrowheads in the insets indicate those of *Math5*-null retina. INL, inner nuclear layer. GCL, ganglion cell layer. ONL, outer nuclear layer. Scale bar equals 100 μm.

To investigate the multiple effects of *Math5*-null mutation, we first assessed the change in different retinal cell types by co-labeling adult control and *Math5*-null retinal sections with cell type-specific markers (Table 1) and quantified the labeled cells separately in three cellular layers. In the GCL of *Math5*-null retinas, there was an increase in BHLHB5+ (GABAergic subtype), PROX1+ (A2 subtype) and GAD65+ (GABAergic subtype) amacrine cells (6-, 2-and 3-fold, respectively; Figure 1A-C, I). The ISL1+ and calretinin+ (ganglion and cholinergic amacrine) cells were reduced by ~50% (Figure 1D, E, I), likely due to the absence of RGCs. Consequently, the total number of PAX6+ cells in the GCL, including all amacrine and ganglion cells, was not significantly changed (Figure 1I and Additional file 1: Supplemental Figure S1A). In the INL, the amacrine cells of GABAergic (GAD65+) subtype were reduced by 29.5% ± 9.2% and of cholinergic (calretinin+) subtype by 34.4% ± 13.4% (Figure 1C, D, I).

**Table 1:** List of antibodies used in this study.

Antibodies	Sources	Working dilution	Retinal cell markers	References
anti-BHLHB5	Santa Cruz Biotech. (Santa Cruz, CA)	1:1,000	displaced amacrine, GABAergic amacrine, and OFF-cone bipolar cells	[16]
anti-BrdU	DSHB (Univ. of Iowa)	1:50	cell cycle, S-phase cells	[16]
anti-BRN3A	Chemicon Intl. (Temecula, CA)	1:400	retinal glanglion cells	[32]
anti-BRN3B	Santa Cruz Biotech. (Santa Cruz, CA)	1:2,000	retinal ganglion cells	[32]
anti-calbindin 28K	Sigma (St. Louis, MO)	1:5,000	horizontal cells, some amacrine cells	[33]
anti-calretinin	Calbiochem (San Diego, CA)	1:2,000	ganglion and amacrine cells of cholinergic, non-AII and displaced subtypes	[33-35]
anti-activated caspase-3	R&D Systems (Minneapolis, MN)	1:200	apoptotic cells	[36]
anti-CHX10	Exalpha (Watertown, MA)	1:200	progenitors, bipolar cells	[37]
anti-GAD65	BD Biosciences (San Jose, CA)	1:200	GABAergic amacrine cells	[38]
anti-Goα	Upstate (Lake Placid, NY)	1:200	ON-bipolar cells	[33]
anti-GFP	MBL (Woaburn, MA) or Abcam, (Cambridge, MA)	1:1,000	green fluoresence protein	[36]
anti-lacZ	DSHB, Univ. of Iowa or Chemicon Intl. (Temecula, CA)	1:500	ß-galactosidase	[36]
anti-ISL1/2	DSHB (Univ. of Iowa)	1:400	retinal ganglion, ON-bipolar, and cholinergic amacrine cells	[39-41]
Anti-Ki67	BD Pharmingen (San Jose, CA)	1:200	cell cycle, all phases	[42]
anti-NEUROD1	Santa Cruz Biotech. (Santa Cruz, CA)	1:500	cone and amacrine cells	[15]
anti-NRL	Chemicon Intl. (Temecula, CA)	1:1,000	rod photoreceptor cells	[43]
anti-p27kip1	BD Biosciences (San Jose, CA)	1:100	Muller cells	[44]
anti-phosphorylated histone 3	Santa Cruz Biotech. (Santa Cruz, CA)	1:400	cell cycle, M-phase	[16]
anti-PAX6	DSHB (Univ. of Iowa)	1:200	progenitors, pan-ganglion, and pan-amacrince cells	[16,45-47]
anti-PKCα	Sigma (St. Louis, MO)	1:5,000	rod bipolar cells	[33]
anti-PROX1	Covance (Berkeley, CA)	1:1,000	progenitors, bipolar, horizintal, AII and displaced amacrince cells	[48]
anti-recoverin	Chemicon Intl. (Temecula, CA)	1:200	Type 2 OFF-cone bipolar, photoreceptor cells	[33,49]
anti-rhodopsin	Chemicon Intl. (Temecula, CA)	1:200	Rod photoreceptor cells	[50]
anti-RXRγ	Santa Cruz Biotech. (Santa Cruz, CA)	1:200	Cone photoreceptor cells	[51]
anti-VSX1	Gift from R.L. Chow (Univ. of Victoria, Canada)	1:100	OFF-cone bipolar cells	[52,53]

In the INL of *Math5*-null retinas, the total number of amacrine (PAX6+) cells was reduced by 21.9% ± 6.2% (Additional file 1: Supplemental Figure S1A) and specifically, a decrease in GAD65+ (29.5% ± 9.2%) and calretinin+ (34.4% ± 13.4%;) subtypes (Figure 1C, D, I). There was also an overall reduction in bipolar cell number (36.7% ± 7.5% less CHX10+ cells; Additional file 1: Supplemental Figure S1B) and specifically, total ON-bipolar cells (Goα+) reduced by 37.1% ± 7.0% (Figure 1I and Additional file 1: Supplemental Figure S1C); ON-rod bipolar cells (PKCα+) by 43.5% ± 11.0%; OFF-cone bipolar cells (VSX1+) by 48.2% ± 3.9%; and recoverin+ Type 2 OFF-bipolar cells by 76.9% ± 11.2% (Figure 1I and Additional file 1: Supplemental Figure S1C, S1D). The reduction of amacrine and bipolar cells in the INL of *Math5*-null retina was also revealed by immunolabeling with anti-BHLHB5 (46.8% ± 9.0% reduction), anti-PROX1 (21.3% ± 3.8% reduction), and anti-ISL1 (19.7% ± 5.4% reduction) (Figure 1A, B, E, I). Interestingly, while all other neuronal cell types in the INL were reduced, the number of horizontal cells (calbinidin+) remained the same in control and *Math5*-null retinas (Figure 1I and Additional file 1: Supplemental Figure S1F). In the ONL, rhodopsin+ and NRL+ rod photoreceptors were reduced by 30.1% ± 5.2% (Figure 1G, 1I, and Additional file 1: Supplemental Figure S1G) whereas RXRγ+ cone photoreceptors were increased by 2-fold in *Math5*-null retinas (Figure 1H, I). Thus, targeted disruption of *Math5 *resulted in significant changes in almost every type of retinal neurons.

### Fate conversion of *Math5*-lineage cells in *Math5*-null retinas

**Figure 2:**
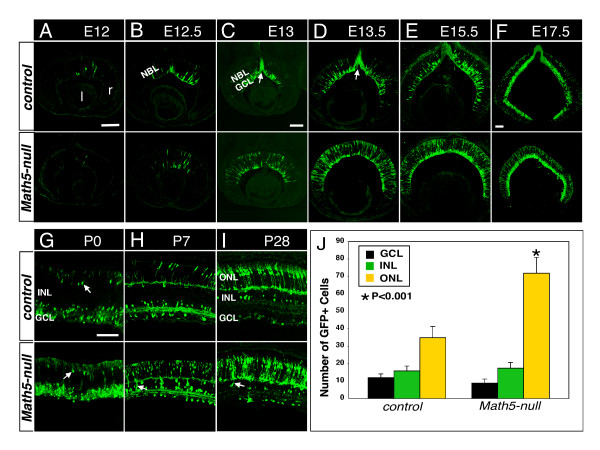
**The spatiotemporal pattern of *Math5*-lineage cells in the developing control and *Math5*-null retinas**. Retinal sections from the control (top panels) and *Math5*-null (bottom panels) retinas at indicated developmental stages were immunolabeled with anti-GFP (green) to mark the *Math5*-lineage cells. (A) The onset of GFP expression starts at E12 in the NBL of the control and *Math5*-null retinas. (B and C) At E12.5-E13, GFP+ cells are present in the NBL, the newly formed GCL, and the RGC axon bundles (arrow) of the control retina. In the *Math5*-null retina no GCL neurons or RGC axon bundles are observed and there are significantly more GFP+ cells in the outermost NBL. (D-F) At E13.5-E17.5, compared to GFP+ cells located in the GCL and the outermost NBL of the control retina, GFP+ cells in the *Math5*-null retina are detected throughout the retina but no visible GFP+ RGC axon bundle are present. Moreover, there is an increase in GFP+ cells in the NBL and a decrease in the GCL. (G) At P0, GFP is expressed in the horizontal-like cells (arrows) in the INL of control and *Math5*-null retinas. (H and I) At P7 and P28, GFP+ cells are located in three retinal layers. Compared to the control, twice as many GFP+ cells were seen in the ONL in *Math5*-null retina and a few GFP+ bipolar-like cells (arrow) are present in the INL. (J) Quantification of GFP+ cells in the ONL, INL and the GCL per 350 μm length of adult retinal sections. l, lens. r, retina. NBL, neuroblast layer. Scale bars equal 100 μm in A (applies to A, B), C (applies to C, D, E), F (applies to F), and G (applies to G-I).

To investigate whether there is a fate conversion of *Math5*-lineage cells in *Math5*-null retinas, we used *Math5-Cre *knock-in mice and *Z/EG *GFP reporter mice to trace the fate of *Math5*-lineage cells. Upon activation, *Z/EG *mice express a conditional GFP reporter gene uniformly in all retinal cell types (Additional file 2: Supplemental Figure S2). First, we compared the distribution of *Math5*-lineage cells in the control (*Math5^Cre/+^*; *Z/EG*) and *Math5*-null (*Math5^Cre/lacZ^*; *Z/EG*) retinas. Anti-GFP labeling showed similar onset of GFP expression in control and *Math5*-null retinas at E12 (Figure 2A), one day after the onset of endogenous *Math5 *expression. From E12.5 to E13.5, while GFP was expressed in the neuroblast layer (NBL), the presumptive GCL, and newly formed RGC axons in control retinas, GFP expression in *Math5*-null retinas were observed in the NBL but not in the GCL nor RGC axons (Figure 2B-D), suggesting the agenesis of RGCs. From E15.5 to E17.5, compared with GFP+ cells seen in the GCL and the presumable cones in the outermost NBL of control retinas, the number of GFP+ cells in *Math5*-null retinas was increased in the NBL and in the outermost NBL but was reduced in the innermost NBL (Figure 2E, F). At P0, GFP+ cells with the morphology resembling horizontal cells could be identified in the INL of the control and *Math5*-null retinas (Figure 2G; arrows). At P7, *Math5*-null retina had more GFP+ cells in the ONL and some GFP+ bipolar-like cells in the INL (Figure 2H; arrow). At P28, GFP+ cells resembling the morphologies of photoreceptors, horizontal, amacrine and ganglion cells were seen in control retinas (Figure 2I). In *Math5*-null retinas, no discernible change in the number of GFP+ cells was observed in the GCL and INL compared to control retina, however, GFP+ cells in the ONL were increased by 2-fold and bipolar-like GFP+ cells (arrow) were observed in the INL (Figure 2I, J).

**Figure 3:**
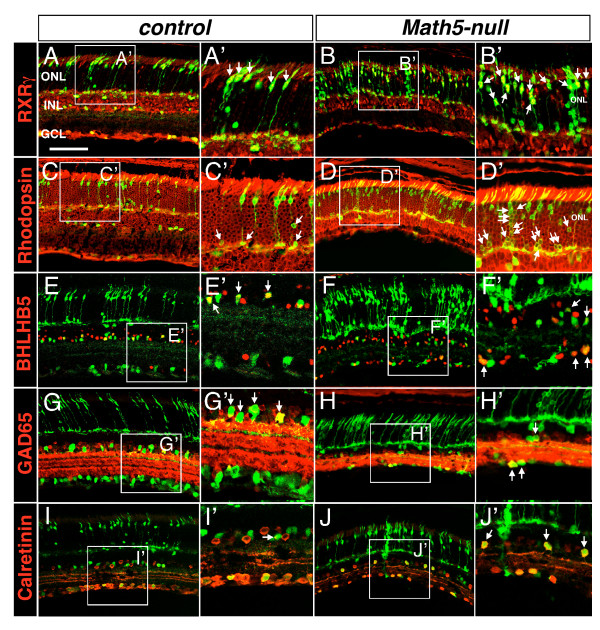
**Fate conversion of *Math5*-lineage cells in *Math5*-null retinas**. Adult retinal sections from the control and *Math5*-null mice were immunolabeled with cell type-specific markers (red) and anti-GFP (green). (A-D) *Math5*-lineage cone photoreceptors marked by RXRγ (arrows in A and B) are increased in number but the rod photoreceptors labeled by rhodopsin (arrows in C and D) are reduced in number in *Math5*-null retinas. (E-H) Among the *Math5*-lineage cells, BHLHB5+ (E, F) and GAD65+ (G, H) GABAergic amacrine cells (arrows) are reduced in number in the INL but are increased in the GCL of *Math5*-null retinas. (I, J) Calretinin+ amacrine subtypes (arrows) are increased in number in the INL of *Math5*-null retinas. Enlarged views of boxed regions are shown in A' to J'. Scale bar equals 100 μm.

**Figure 4:**
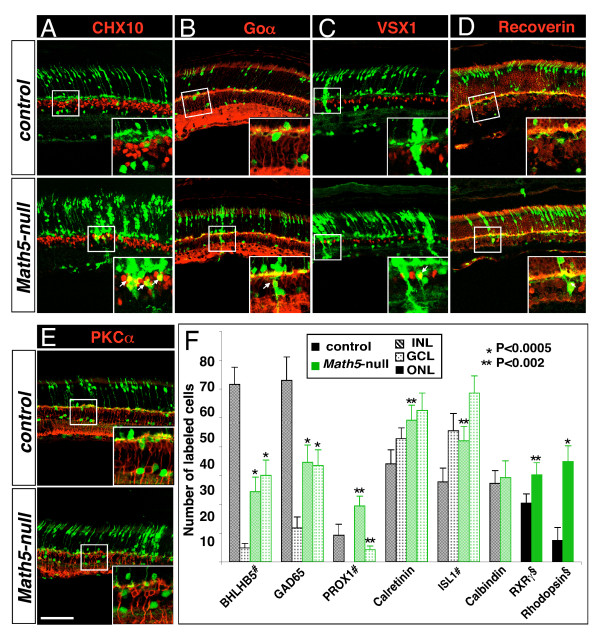
**The cone-bipolar fate of *Math5*-lineage cells in the absence of *Math5***. Adult retinal sections from the control (top panels) and *Math5*-null (bottom panels) mice were immunolabeled with cell type-specific markers (red) and anti-GFP (green). (A-E) Compared to the control retina in which GFP expression is not co-localized with bipolar cell markers, a few of GFP+ cells in the *Math5*-null retina are positive for CHX10 (arrows), Goα (arrow), VSX1 (arrow) or recoverin (arrow) but not for PKCα (E). (K) Quantification of GFP+ cells expressing specific markers in the ONL, INL and the GCL per retinal section. Scale bar equals 100 μm. Notes: #For BHLHB5+, PROX1+ and ISL1+ cells in the INL, only the amacrine cells were counted per retinal section; §RXRγ+ and rhodopsin+ cells were counted per 350 μm linear length.

To determine the identities of *Math5*-lineage cells in the ONL, we immunolabeled retinas with anti-RXRγ and anti-rhodopsin for cone and rod cells, respectively. In *Math5*-null retinas, the cones and rods of *Math5*-lineage (GFP+) were increased by 1.5-and 4-fold, respectively (Figure 3A-D, 4F, and Additional file 3: Supplemental Figure S3A). Within the INL, anti-PAX6 labeling did not detect any overt change in the total number of *Math5*-lineage amacrine cells in the absence of *Math5 *(Additional file 3: Supplemental Figure S3B). However, *Math5*-lineage GABAergic amacrine cells (BHLHB5+ or GAD65+) were significantly reduced in *Math5 *mutants (Figure 3E-H, 4F). In contrast, there was an increase of *Math5*-lineage A2 (PROX1+), cholinergic (ISL1+) and calretinin+ amacrine cell subtypes in *Math5*-null compared to control (Figures 3I, 3J, 4F and Additional file 3: Supplemental Figure S3 D and S3C). In the GCL of *Math5*-null retinas, there was an increase in displaced amacrine cells (BHLHB5+ and GAD65+) of *Math5*-lineage (Figure 3E-H, 4F). In addition, anti-PROX1 labeled GFP+ displaced amacrine cells were found in the GCL of *Math5*-null retinas, which were not detected in control retinas (Figure 4F and Additional file 3: Supplemental Figure S3C).

In control retinas, *Math5*-lineage cells were not observed in bipolar cells but loss of *Math5 *led to the appearance of bipolar cells expressing CHX10 and GFP (Figure 4A). Interestingly, these cells expressed Goα, Vsx1, or recoverin (Figure 4B-D) but not PKCα (Figure 4E), demonstrating that a small percentage of *Math5*-lineage cells switch their fate to cone-bipolar but not to rod-bipolar cells in the absence of *Math5*. Moreover, compared to the absence of Müller cells originating from *Math5*-lineage in the control, loss of *Math5 *also resulted in a very small number of *Math5*-lineage cells adopting the fate of Müller cells (p27kip1+/GFP+; Additional file 3: Supplemental Figure S3F). Overall, we observed no change in the total number of horizontal cells (Figure 4F and Additional file 3: Supplemental Figure S3E).

### Changes in cell proliferation and apoptosis in *Math5*-null retinas

**Figure 5:**
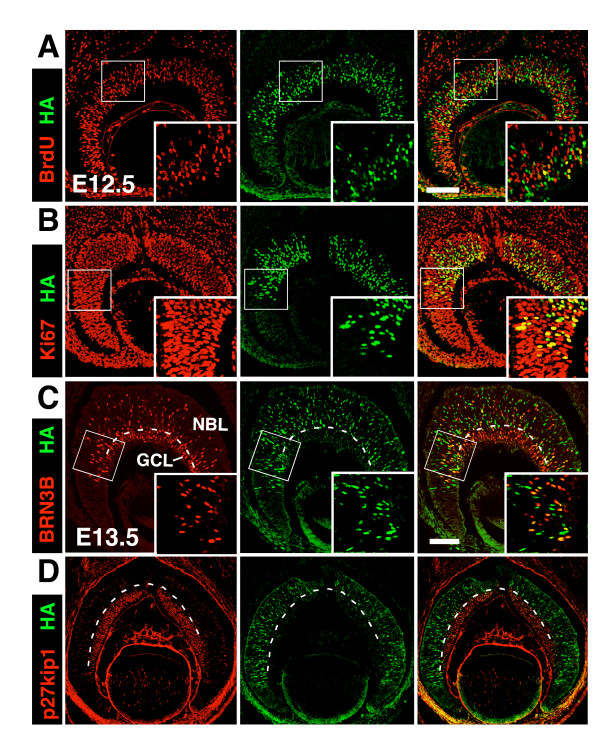
**Expression of MATH5 in retinal progenitors and migrating RGCs**. Retinal sections from the *Math5*-*HA *embryos at the indicated developmental stages were immunolabeled with anti-HA (green) to detect MATH5 and cell type-specific markers (red). (A and B) Partial co-localization of HA with BrdU (A) for mitotic cells at S-phase and Ki67 (B) for all mitotic cells reveals the expression of MATH5 is some progenitors at E12.5. (C and D) The expression of MATH5 is largely restricted in the NBL but not the newly formed GCL marked by BRN3B (C) and p27kip1 (D) expression at E13.5. Colabeling of HA and BRN3B shows the partial co-localization of MATH5 and BRN3B in the nascent, migrating RGCs in the NBL. Boxed regions are shown in the inserts. Dashed line is the arbitrary border between the GCL and the NBL. Scale bars are 100 μm.

Due to the lack of working anti-MATH5 antibody, it remains unknown whether MATH5 is expressed in progenitors or postmitotic cells. We genetically tagged MATH5 by fusing a HA-tag in-frame to MATH5's C-terminus using the knock-in approach in mice and used anti-HA to monitor MATH5 expression. The homozygous *Math5^HA/HA ^*mice were normal and had retinas indistinguishable from their wild type controls (data not shown), indicating that the HA-tag does not interfere with the function of MATH5. Co-immunolabeling of *Math5-HA *retinas at E12.5 with anti-HA and cell cycle markers demonstrated MATH5's expression in the NBL where it partially overlapped with BrdU and Ki67 (Figure 5A,B), indicating that some MATH5+ cells are proliferating progenitors. When the GCL is clearly identifiable at E13.5, labeling of *Math5-HA *retinas showed that MATH5 expression was mostly detected in the NBL where it partially colocalized with BRN3B, but was absent in the newly formed GCL where BRN3B and p27kip1 were highly expressed (Figure 5C, D). Thus, our data indicate that MATH5 is expressed in retinal progenitors as well as in the nascent, migrating RGCs but its expression is turned off in the post-migration RGCs in the GCL of the early retina.

**Figure 6:**
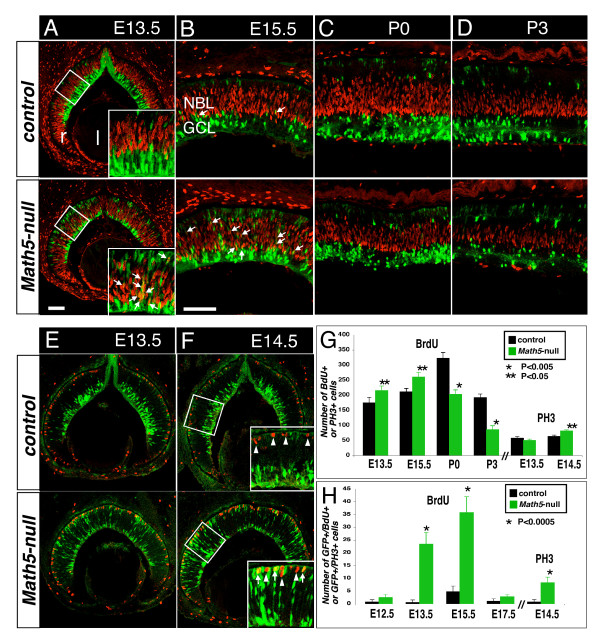
**Loss of *Math5 *alters the proliferation of retinal progenitors and causes the increased *Math5*-lineage cells in cell cycle**. Retinal sections from the control (top panels) and *Math5*-null (bottom panels) mice at the indicated developmental stages were immunolabeled with cell proliferation markers (red) BrdU (S-phase) or PH3 (M-phase) and anti-GFP (green). (A, B) At E13.5 to E15.5, GFP+/BrdU+ cells (arrows) are rarely detected in control retinas but are frequently seen in *Math5*-null retinas. (C, D) At P0 and P3, no visible GFP+/BrdU+ cells are found in the control and *Math5*-null retina, and the BrdU+ cells are profoundly decreased in *Math5*-null retinas. (E and F) PH3+ cells (red; arrowheads) are rarely seen as GFP+ cells in the control retina but in *Math5*-null retina, the GFP+/PH3+ cohorts (arrows) are significantly increased. (G) Quantification of the total BrdU+ or PH3+ cells per 350 μm length. (H) Quantification of the GFP+/BrdU+ or GFP+/PH3+ cells per retinal section. Scale bar equals 100 μm in A (applies to A, E and F), B (applies to B-D).

A recent study has shown that MATH5 promotes cell cycle exit of retinal progenitors in E12.5 mouse retinal explants [13]. To test whether *Math5*-null mutation affects the cycle exit of *Math5*-lineage cells, we pulse-labeled the developing control and *Math5*-null retinas with BrdU. While anti-BrdU labeling showed a 23.8% ± 9.6% increase of total mitotic cells at S-phase in *Math5*-null retinas at E13.5 and 23.2% ± 6.2% at E15.5, the BrdU+ cells of *Math5*-lineage (GFP+) were increased by 40-fold at E13.5 and 7.5-fold at E15.5 (Figure 6A, B, G, H). Similarly, anti-phosphorylated histone 3 (PH3) revealed a 28.4% ± 6.3% increase in all mitotic cells at M-phase in *Math5*-null retina at E14.5 and GFP+ cells at M-phase were increased by 10.5-fold (Figure 6E-H). There was no significant change in the total number of proliferating cells at E12.5 and E17.5 (Figure 6G, 6H, and Additional file 4: Supplemental Figure S4). In postnatal retinas, loss of *Math5 *resulted in a reduction in the number of BrdU+ cells by 37.1% ± 5.1% at P0 and 55.1% ± 6.4% at P3 (Figure 6C, D, G, H).

**Figure 7:**
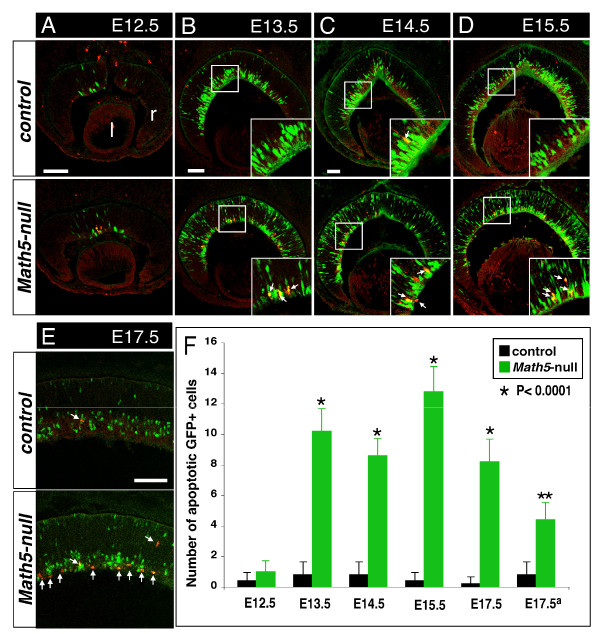
***Math5*-lineage cells undergo apoptosis in *Math5*-null retinas**. Retinal sections from the control (top panels) and *Math5*-null (bottom panels) embryos at the indicated developmental stages were immunolabeled with anti-activated caspase-3 (red) and anti-GFP (green). (A-E) At E12.5 to E17.5, compared to the GFP+ apoptotic cells (arrows) occasionally seen in control retinas, the number of GFP+ apoptotic cells is significantly increased in *Math5*-null retinas. Insets in B-D indicate the enlarged views of boxed regions. Scale bar equals 100 μm. (F) Quantification of the GFP+/caspase-3+ cells per retinal section. Note: *a*, apoptotic cells negative for GFP were counted.

Additionally, we examined the change in apoptosis of *Math5*-lineage cells by co-immunolabeling with anti-GFP and anti-activated caspase-3. Compared to a low number of apoptotic cells of *Math5*-lineage in control retinas, loss of *Math5 *resulted in a significant increase in apoptosis of *Math5*-lineage cells at E12.5 to E17.5 (Figure 7) but no overt change was found after P0 (data not shown). Moreover, a majority of the apoptotic *Math5*-lineage cells were located in the innermost layer of developing retinas, the presumable GCL. Thus, the change in proliferation and apoptosis could contribute to the broad effect of *Math5*-null mutation on multiple retinal cell types.

### Premature expression of non-RGC regulator proteins in *Math5*-null retinas

**Figure 8:**
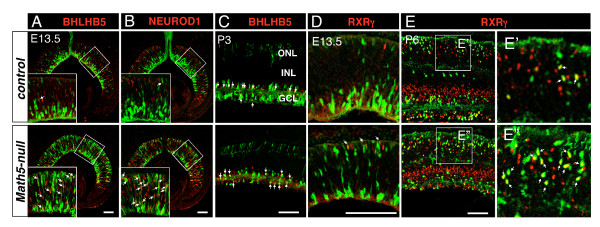
**Loss of *Math5 *leads to an up-regulated expression of non-RGCs factors in *Math5*-lineage cells**. Retinal sections from the control (top panels) and *Math5*-null (bottom panels) embryos at the indicated developmental stages were immunolabeled with cell type markers (red) and anti-GFP (green). (A, B) At E13.5, many GFP+ *Math5*-lineage cells express BHLHB5 (A) or NEUROD1 (B) (arrows) in the *Math5*-null retina but not in the control retina. (C) At P3, compared to the control, more GFP+/BHLHB5+ cells (arrows) are seen in the GCL and less in the INL of *Math5*-null retinas. (D, E) The number of GFP+/RXRγ+ cone cells (arrows) is increased in *Math5*-null retinas at E13.5 (D) and P6 (E). Enlarged view of boxed regions in E is shown in E'. Scale bars equal 100 μm.

To investigate the molecular mechanisms underlying the increased number of displaced amacrine cells in *Math5*-null retina, we analyzed the expression of NEUROD1 and BHLHB5, two essential molecules in the genesis of photoreceptor and amacrine cells [14-16]. Compared to the expression of BHLHB5 and NEUROD1 in a very few *Math5*-lineage cells at E13.5 in control retinas, there was a significant increase in *Math5*-lineage cells expressing BHLHB5 and NEUROD1 in *Math5*-null retinas (Figure 8A, B). Such an increase was first detected at E12.5, the earliest stage when *Math5*-lineage cells could be identified by the Cre-loxP approach (Additional file 5: Supplemental Figure S5A, B). At E17.5, anti-BHLHB5 labeling marks cells mostly located at the opposing borders of the GCL and the developing INL (Additional file 5: Supplemental Figure S5C), these are amacrine cells. In the absence of *Math5*, RGCs were rarely detected by anti-BRN3B labeling (Additional file 5: Supplemental Figure S5D), while more BHLHB5+ cells of *Math5*-lineage were seen in the GCL (Additional file 5: Supplemental Figure S5C). This change was more obvious at postnatal stages (Figure 8C, arrows). Similarly, more amacrine cells were identified by anti-ISL1 labeling (Additional file 5: Supplemental Figure S5E), but not by anti-calretinin that marks the late-born amacrine cells (Additional file 5: Supplemental Figure S5F).

We also analyzed the expression of NEUROD1 and RXRγ that are required for the genesis of photoreceptors. At E17.5, many NEUROD1+ cells were located near the outermost NBL in both control and *Math5*-null retinas. The location of these cells suggests that they are photoreceptor cells (Additional file 5: Supplemental Figure S5G). Co-labeling the retinas with anti-RXRγ (cone marker) at E13.5, E17.5, and P6 demonstrated that many of the additional GFP+ photoreceptors are in fact cone photoreceptors of *Math5*-lineage (Figure 8D, 8E, and Additional file 5: Supplemental Figure S5H).

## Discussion

In this study, we have used the Cre/loxP approach to trace and compare the fates of *Math5*-lineage cells in normal and *Math5*-null retinas. The results presented here demonstrate that in the absence of *Math5*, there is an increase of *Math5*-lineage cells adopting amacrine, cone, and rod cell fates or choosing the new fates of cone bipolar and Müller cells. Furthermore, the loss of *Math5 *has a broad effect on retinal development including the development of multiple retinal cell types, cell proliferation, and cell survival.

### Role of MATH5 in regulating multiple retinal cell fates

Cell lineage tracing studies have revealed that all retinal neurons and Müller cells are derived from a common pool of progenitors, and the daughter cells of a progenitor often assume distinct neuronal identities [17-20]. These studies suggest that cell fate decisions are made during or after the terminal neurogenic cell cycle under the influence of environmental cues. Subsequent studies have shown that transcription factors play pivotal roles in the specification and differentiation of distinct retinal cell types [8,21-23]. Thus, the interplay between cell-extrinsic cues and cell-intrinsic factors is critical for the outcome of retinal cell differentiation.

Previous analysis of *Math5*-lineage cells during normal retinal development reveals that *Math5 *is expressed in retinal progenitors giving rise to all horizontal and ganglion cells and to some amacrine and photoreceptor cells [12]. Nevertheless, it is not well understood whether the development of other retinal cells is affected and whether there is a fate conversion of *Math5*-lineage cells in the absence of *Math5*. Using lineage tracing analysis, we have found that in the INL of *Math5*-null retinas, there is a reduced number of GABAergic amacrine cells accompanied by an increase in cholinergic and A2 glycinergic amacrine cells from *Math5*-lineage (Figure 3). Similarly, in the GCL of *Math5*-null retinas, the loss of RGCs goes together with an increase of the displaced amacrine cells from *Math5*-lineage, and more rod and cone cells arise from *Math5*-lineage in the ONL of *Math5*-null retinas (Figures 3, 4 and Additional file 3: Supplemental Figure S3). All of these changes clearly demonstrate cell fate switch in *Math5*-lineage cells after the ablation of *Math5*. Moreover, we have observed a small number of *Math5*-lineage cells adopting the fate of cone bipolar cells and Müller cells in *Math5*-null retinas despite that these retinal cell types are generated postnatally during normal retinal development (Figure 4 and Additional file 3: Supplemental Figure S3).

**Figure 9:**
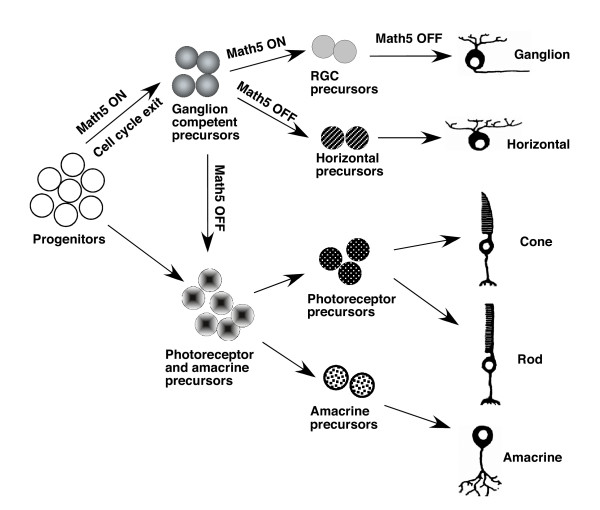
**A model for the role of MATH5 in the sequential formation of early-born retinal neurons**. During normal retinogenesis, the expression of MATH5 starts in selected retinal progenitors and promotes the cell cycle exit. The transient expression of MATH5 makes the postmitotic precursors become RGC-competent and temporarily blocks non-RGC differentiation programs by suppressing the expression of retinogenic factors such as NEUROD1, NGN2, MATH3, BHLHB5, and RXRγ. During this competence period, MATH5 activates a network of transcription factors including BRN3B and ISL1 in some precursors to initiate the RGC differentiation program. The remaining precursors lose RGC-competence when MATH5 expression ceases, express other retinogenic factors and generate horizontal cells (all horizontal cells are of *Math5*-lineage), or along with the non-*Math5*-lineage precursors, produce amacrine, rod, and cone cells.

The fate conversion of *Math5*-lineage cells from RGCs to other retinal neurons and Müller glial cells in *Math5*-null mice demonstrated here, extends previous loss-of-function analysis of *Math5 *and suggests that MATH5 directly regulates the acquisition of multiple cell fates by retinal progenitors. MATH5 not only promotes RGC fate but also suppresses the fate choices of amacrine, cone, rod, cone bipolar, and Müller cells. We analyzed the expression of NEUROD1, RXRγ, and BHLHB5 that are implicated in the development of cones, pan-amacrine cells and the subset of GABAergic amacrine cells, respectively [15,16]. Interestingly, the fate conversion of *Math5*-lineage cells is associated with the precocious expression of these transcription factors (Figure 8). Since *Math5*-lineage cells give rise to RGC and other retinal neurons during normal development [12], MATH5 functions as a RGC competence factor and it alone is insufficient to specify RGC fate. We hypothesize that during normal retinogenesis, the transient MATH5 expression provides progenitors with a brief window of opportunity to choose RGC fate as the first choice (Figure 9). During this RGC competence period, MATH5 transiently suppresses the differentiation program of selected retinal neurons by directly or indirectly regulating *Neurod1*, *Bhlhb5*, and *RXRγ *and possibly interacting with other non-RGC regulators such as *Foxn4*. Through this mechanism, MATH5 influences cell fate choice and determines birth order of retinal cells that RGCs are generated first. Likewise, the cell fate switch of *Math5*-lineage cells from RGC to cone-bipolar cells could result from the up-regulation of BHLHB5 as BHLHB5 is associated with the genesis of a subset of off-cone bipolar cells [16].

Though cell birthdating experiments show that retinal neurons are generally produced in two phases: ganglion, horizontal, amacrine, and cone cells are generated during the early embryonic phase, and rod, bipolar and Müller cells are produced in the later postnatal phase [3]. It is not clear whether the early embryonic progenitors in the early environment are competent to differentiate into later born retinal cells. Surprisingly, we have observed that in *Math5*-null retinas, an increased number of *Math5*-lineage cells are converted into the late born rod, cone bipolar and Müller cells (Figure 4 and Additional file 3: Supplemental Figure S3). Previous birthdating studies in *Math5*-null retinas have also shown that some Müller cells are born precociously as early as at E11.5 [24]. Taking together, these findings suggest that the early retinal environment could permit the differentiation of late-born retinal cells but intrinsic factors, such as MATH5, suppress progenitors' potential to differentiate into these late born retinal cells. Future birthdating experiments of *Math5*-lineage cells in *Math5*-null retinas will confirm this possibility. Additionally, the cell fate conversion of *Math5*-lineage cells into cone bipolar cells in *Math5*-null retinas suggests that the differentiation of specific bipolar subtypes might require different intrinsic and or environment factors. Alternatively,

### The role of Math5 in cell cycle

The multiple retinal cell fates of *Math5-*lineage cells imply that these *Math5*-expressing cells are not yet committed to a particular cell fate. We found that in the absence of *Math5*, the number of proliferating retinal progenitors is transiently increased at E13.5 to E15.5 but is reduced later in the first postnatal week (Figure 6). The postnatal drop in cell proliferation rate could be caused by the loss of RGCs in *Math5*-null retinas since SHH produced by the early born RGCs are required to maintain the proliferation of the late retinal progenitors and the loss of RGCs results in down-regulation of GLI1, a SHH effector, in retinal progenitors [25]. It is likely that loss of *Math5 *leads to the reduced expression of GLI1, which in turn, could account for the reduced proliferation later. The early transient increase in cell proliferation in *Math5*-null retinas is consistent with the previous report [24]. However, it remains uncertain whether MATH5 plays a role in driving the progenitors to exit cell cycle and whether *Math5*-lineage cells remain in or re-enter cell cycle without MATH5. Using lineage analysis, we have observed that in the absence of *Math5*, the number of mitotic *Math5*-lineage cells is significantly increased (Figure 6) at E13.5 to E15.5. In a recent study, the ectopic expression of *Math5 *and another neurogenic gene *Ngn3 *in E12.5 mouse retinal explants leads to a significant reduction in the number of progenitors in BrdU-labeled S-phase [13]. Thus, it is likely that a key role of MATH5 in retinal development is to drive the cell cycle exit of progenitors. Without MATH5, the retinal progenitors continue proliferating or delay the cell cycle exit.

Previous studies showed that *Math5 *mRNA expression is largely restricted in the NBL, suggesting its expression in progenitors [12,26]. However, studies using *Math5*-lacZ reporter mice revealed a largely postmitotic expression pattern of lacZ, implying *Math5 *expression after the cell cycle exit of progenitors [12,24]. These contradicting observations likely reflect the transient nature of *Math5 *mRNA and the long half-life of lacZ reporter protein. To resolve this issue, we have genetically tagged endogenous MATH5 with HA-tag. Colocalization of HA-tag and cell cycle markers reveals significant more mitotic progenitors expressing MATH5 than previously reported using the Math5-lacZ reporter. One possible explanation of this apparent discrepancy is that lacZ protein is more stable than MATH5 and persists in postmitotic, differentiated cells, such as the newly formed RGCs [12,24]. On the other hand, MATH5 expression, revealed by the HA-tag, is likely highly transient and mirrors the dynamic mRNA expression pattern in cells of the NBL but not in the GCL [12,26]. The observation of *Math5*-lacZ expression mostly in postmitotic RGCs of the GCL and of MATH5-HA in cells of the NBL further demonstrates that the onset of *Math5 *expression begins in progenitors in the NBL and that MATH5 expression drives progenitors to exit cell cycle and to differentiate into RGCs and other retinal neurons.

Our results have also revealed another function of MATH5 in cell survival. Based on the increased apoptotic cells from *Math5*-lineage in *Math5*-null embryonic retinas, MATH5 could suppress the apoptosis of retinal cell types during normal embryogenesis. The spatiotemporal pattern of these apoptotic cells suggests their identities as RGCs and amacrine cells because most of these cells are located near the innermost region (the presumptive GCL) of *Math5*-null retinas and are found soon after the activation of lineage reporter GFP (Figure 6). Future experiments are needed to address how MATH5 regulates the apoptosis. Nevertheless, it is likely that the apoptosis partially accounts for the reduced cell numbers of RGCs or amacrine cells in adult *Math5*-null retina.

## Materials and methods

### Animals

The *Math5*-*lacZ *and *Math5*-*Cre *knock-in mice were generated previously in our laboratory [12,27]. *CMV-ß-actin-Cre *[28], *R26R-lacZ *[29], *xstpx-lacZ *[30], and *Z/EG *[31] mice were obtained from The Jackson Laboratory (Bar Harbor, Maine). PCR genotyping of the reporter mice was performed according to protocols provided by The Jackson Laboratory. Embryos were identified as E0.5 at noon on the day at which vaginal plugs were first observed. All animal experiments performed in this study were approved by the University Committee of Animal Resources (UCAR) at University of Rochester.

### Immunohistochemistry

Staged mouse embryos were dissected and fixed in 4% paraformaldehyde (PFA) in PBS at 4°C for 1-2 hours. Isolated postnatal and adult retinas were fixed for 30 minutes. Fixed samples were saturated in 20% sucrose in PBS and embedded in Tissue-Tek O.C.T. (Sakura, Torrance, CA) for cryosections cut at a thickness of 14 μm. Immunofluorescent labeling and BrdU pulse-labeling experiments were performed as described [12]. Working dilutions and sources of antibodies used in this study are listed in Table 1. Alexa-conjugated secondary antibodies were obtained from Molecular Probes (Eugene, OR) and were used at a concentration of 1:1,000. Images were digitally captured using a Zeiss 510 META confocal microscope. To quantify immunolabeled retinal cells, the number of cells per retinal section or within 350 μm field length was counted for each retina and at least five retinas were averaged for each cell type. All results were analyzed for student's t-test significance using Microsoft Excel program.

## Competing interests

The authors declare that they have no competing interests.

## Authors' contributions

LF writes the manuscript and is responsible for all figures except Figure 5. ZX and QD are responsible for Figure 5. XX and LG are responsible for generating *Math5*-HA mice. LG is responsible for experimental design. LG and RL revise the manuscript. All authors read and approved the final manuscript.

## Supplementary Material

Additional file 1 **Supplemental Figure S1. Changes of retinal cell subtypes in *Math5*-null retinas**. Retinal sections from the control (top panels) and *Math5*-null (bottom panels) mice at P28 were immunolabeled with cell type-specific markers (green) and nuclear counterstained with propidium iodide (PI, red). PAX6+ amacrine cells in the INL (A), CHX10+ bipolar cells (B), Goα+ ON-bipolar cells (C), PKCα+ rod bipolar cells (D), recoverin+ Type 2 OFF-cone bipolar cells (E) and NRL+ rod photoreceptors (G) are reduced in number in *Math5*-null retina. No overt change is seen in calbindin+ horizontal cells (F). Scale bar equals 100 μm. Click here for file

Additional file 2 **Supplemental Figure S2. Lineage analysis of *Math5*-expressing cells**. (A) Schematic description of the Cre/loxP mediated conditional activation of reporter genes using *Math5*-Cre and *lacZ *or *EGFP *reporter mouse lines. (B-D) Expression comparison of three reporter genes in retinas. Adult retinal sections from three different reporter lines, *CMV-Cre/+; R26R-lacZ, CMV-Cre/+; xstpx-lacZ *and *CMV-Cre/+; Z/EG *were immunolabeled with anti-lacZ or anti-GFP (green). In contrast to the biased expression of lacZ expression in the cells of the GCL and the INL in *R26R *and *xstpx-lacZ *mice (B and C), the GFP expression in the *CMV-Cre/+; Z/EG *retina reveals a uniform distribution of GFP+ cells in all retinal cells (D). Scale bar equals 100 μm. Click here for file

Additional file 3 **Supplemental Figure S3. Line Analysis of retinal cell types from *Math5*-lineage**. Retinal sections from the indicated developmental stages were immunolabeled with cell type/proliferation markers (red) and anti-GFP (green). There was no discernible change in the number of PAX6+/GFP+ amacrine cells in the INL within adult *Math5*-null retina (B), while there was an increase in NRL+/GFP+ rods, ISL1+/GFP+ cells, PROX1+/GFP+ amacrine cells, and PROX1+/GFP+ displaced amacrine cells (C and D). No overt change was detected in calbindin+/GFP+ and p27kip1+ cells (E and F). Scale bar is 100 μm. Click here for file

Additional file 4 **Supplemental Figure S4. Comparison of cell proliferation rate in the normal and *Math5*-null retinas**. Retinal sections from the indicated developmental stages were immunolabeled with cell proliferation markers BrdU or PH3 (red) and anti-GFP (green). The number of GFP+/BrdU+ and GFP+/PH3+ proliferating cells in *Math5*-null retinas is comparable to that in the controls at E12.5 and E17.5. Scale bar equals 100 μm. Click here for file

Additional file 5 **Supplemental Figure S5. Altered expression of RGCs factors and non-RGCs factors in the developing ***Math5***-lineage cells in the absence of *Math5***. Retinal sections from the indicated developmental stages were immunolabeled with cell type markers (red) and anti-GFP (green). At E12.5, GFP+/BHLHB5+ or GFP+/NEUROD1+ cells are rarely detected in the control retina, whereas their cohorts are seen in the *Math5*-null retina (A and B). At E17.5, more GFP+/BHLBHB5+ cohorts are seen in the GCL as well as GFP+/NEUROD1+ cohorts in the outermost NBL in the *Math5*-null retina (C and G). While fewer GFP+/BRN3B+, GFP+/ISL1+, and GFP+/calretinin+ cohorts are detected in the *Math5*-null retina (D-F), the GFP+/RXRγ+ cells are increased in number in the outermost NBL (H). Enlarged views of boxed regions in A and B are shown, respectively. Scale bars equal 100 μm in A (applies to A and B), C (applies to C-H). Click here for file
