# Supplementary figures and images for: MATH5 controls the acquisition of multiple retinal cell fates
Source: Mol Brain. 2010 Nov 18;3:36. doi: 10.1186/1756-6606-3-36 (PMC2994854; doi:10.1186/1756-6606-3-36)

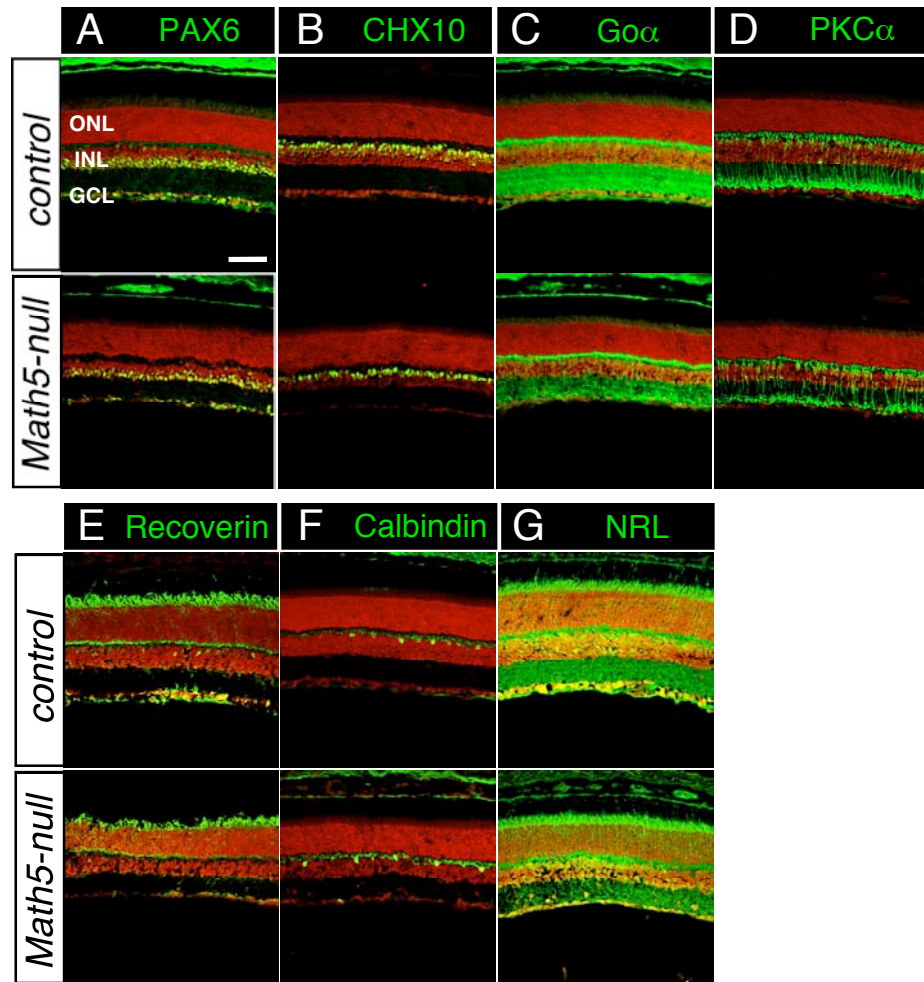

Supplement: Additional file 1 — Supplemental Figure S1. Changes of retinal cell subtypes in Math5-null retinas. Retinal sections from the control (top panels) and Math5-null (bottom panels) mice at P28 were immunolabeled with cell type-specific markers (green) and nuclear counterstained with propidium iodide (PI, red). PAX6+ amacrine cells in the INL (A), CHX10+ bipolar cells (B), Goα+ ON-bipolar cells (C), PKCα+ rod bipolar cells (D), recoverin+ Type 2 OFF-cone bipolar cells (E) and NRL+ rod photoreceptors (G) are reduced in number in Math5-null retina. No overt change is seen in calbindin+ horizontal cells (F). Scale bar equals 100 μm. [file 1756-6606-3-36-S1.PDF]

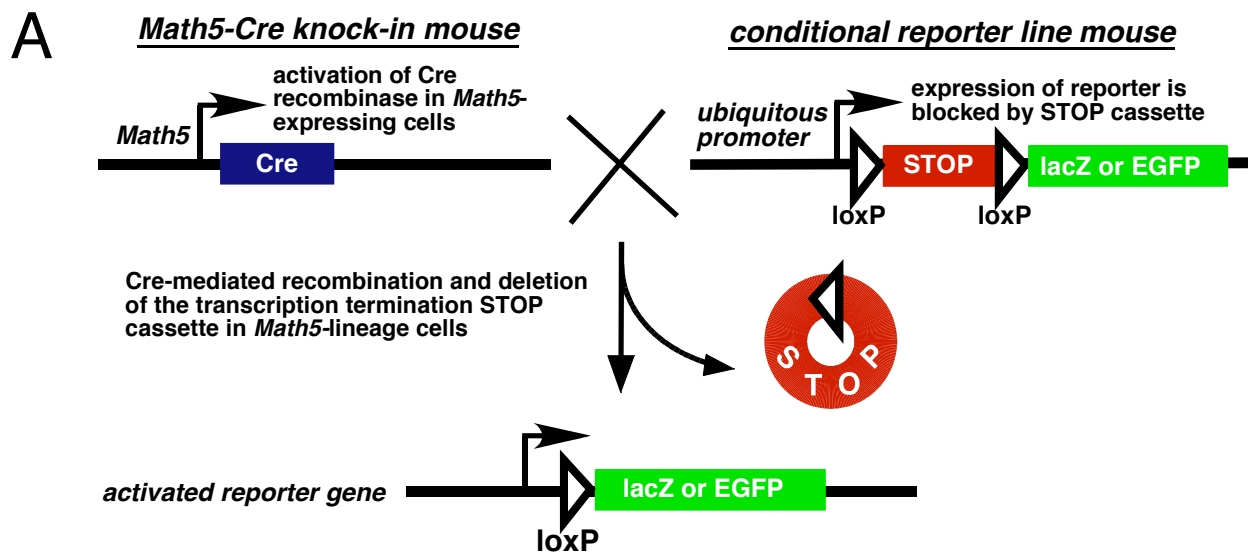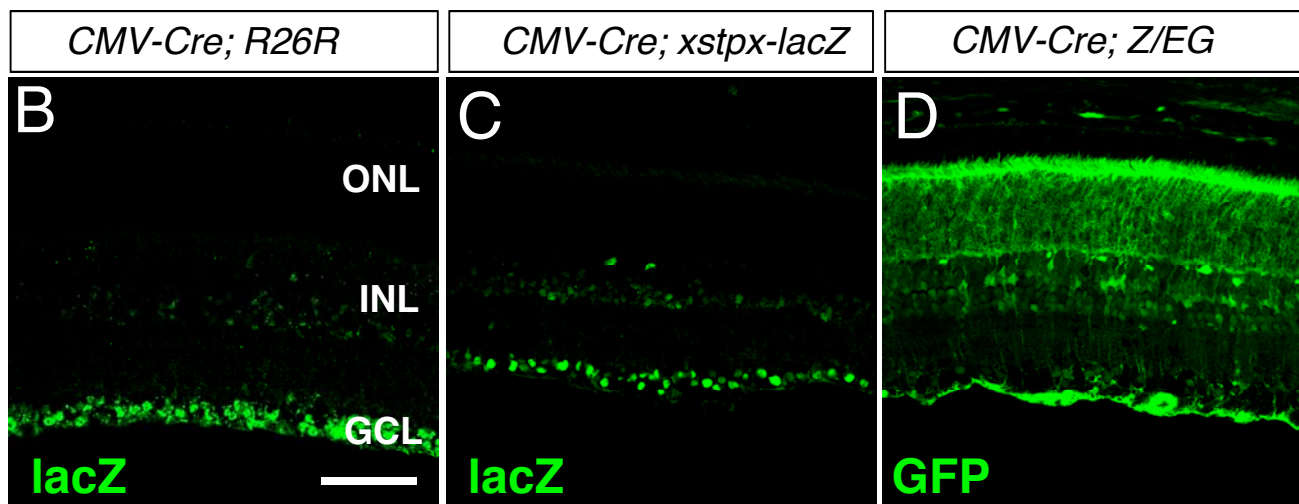

Supplement: Additional file 2 — Supplemental Figure S2. Lineage analysis of Math5-expressing cells. (A) Schematic description of the Cre/loxP mediated conditional activation of reporter genes using Math5-Cre and lacZ or EGFP reporter mouse lines. (B-D) Expression comparison of three reporter genes in retinas. Adult retinal sections from three different reporter lines, CMV-Cre/+; R26R-lacZ, CMV-Cre/+; xstpx-lacZ and CMV-Cre/+; Z/EG were immunolabeled with anti-lacZ or anti-GFP (green). In contrast to the biased expression of lacZ expression in the cells of the GCL and the INL in R26R and xstpx-lacZ mice (B and C), the GFP expression in the CMV-Cre/+; Z/EG retina reveals a uniform distribution of GFP+ cells in all retinal cells (D). Scale bar equals 100 μm. [file 1756-6606-3-36-S2.PDF]

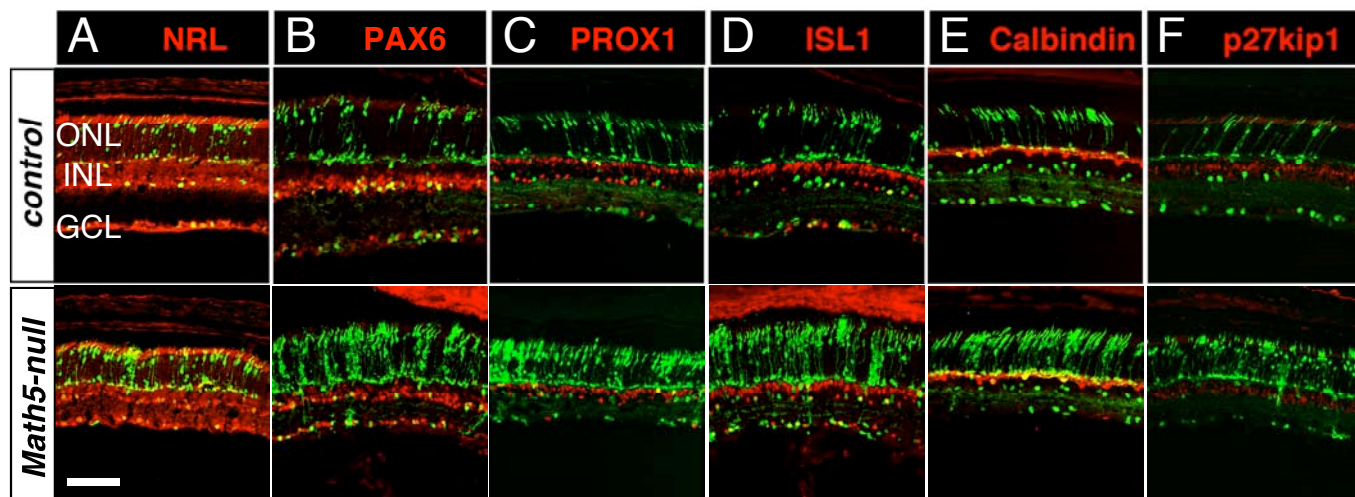

Supplement: Additional file 3 — Supplemental Figure S3. Line Analysis of retinal cell types from Math5-lineage. Retinal sections from the indicated developmental stages were immunolabeled with cell type/proliferation markers (red) and anti-GFP (green). There was no discernible change in the number of PAX6+/GFP+ amacrine cells in the INL within adult Math5-null retina (B), while there was an increase in NRL+/GFP+ rods, ISL1+/GFP+ cells, PROX1+/GFP+ amacrine cells, and PROX1+/GFP+ displaced amacrine cells (C and D). No overt change was detected in calbindin+/GFP+ and p27kip1+ cells (E and F). Scale bar is 100 μm. [file 1756-6606-3-36-S3.PDF]

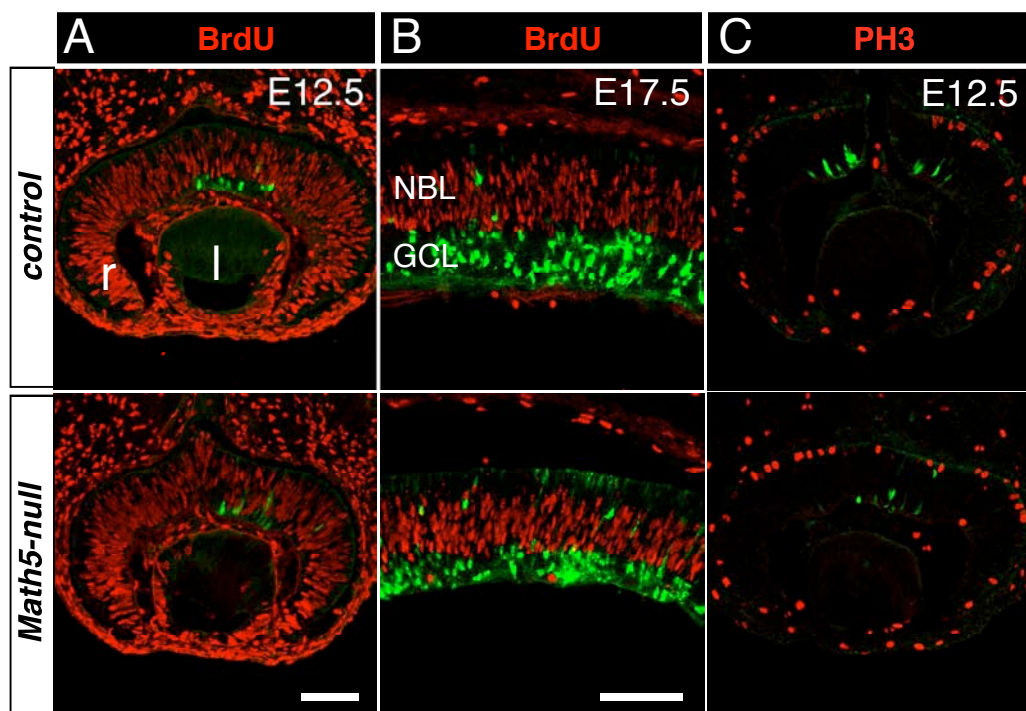

Supplement: Additional file 4 — Supplemental Figure S4. Comparison of cell proliferation rate in the normal and Math5-null retinas. Retinal sections from the indicated developmental stages were immunolabeled with cell proliferation markers BrdU or PH3 (red) and anti-GFP (green). The number of GFP+/BrdU+ and GFP+/PH3+ proliferating cells in Math5-null retinas is comparable to that in the controls at E12.5 and E17.5. Scale bar equals 100 μm. [file 1756-6606-3-36-S4.PDF]

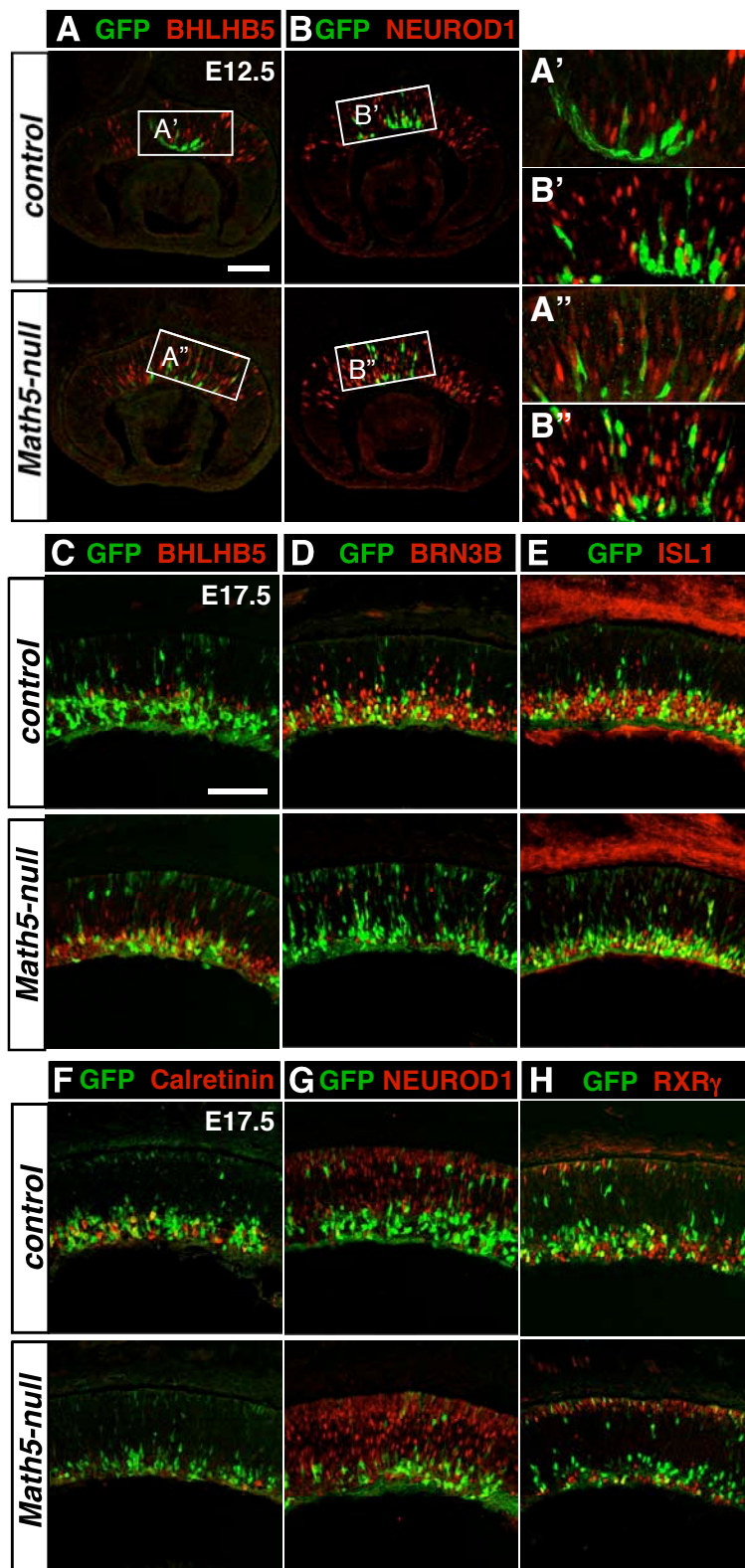

Supplement: Additional file 5 — Supplemental Figure S5. Altered expression of RGCs factors and non-RGCs factors in the developing Math5-lineage cells in the absence of Math5. Retinal sections from the indicated developmental stages were immunolabeled with cell type markers (red) and anti-GFP (green). At E12.5, GFP+/BHLHB5+ or GFP+/NEUROD1+ cells are rarely detected in the control retina, whereas their cohorts are seen in the Math5-null retina (A and B). At E17.5, more GFP+/BHLBHB5+ cohorts are seen in the GCL as well as GFP+/NEUROD1+ cohorts in the outermost NBL in the Math5-null retina (C and G). While fewer GFP+/BRN3B+, GFP+/ISL1+, and GFP+/calretinin+ cohorts are detected in the Math5-null retina (D-F), the GFP+/RXRγ+ cells are increased in number in the outermost NBL (H). Enlarged views of boxed regions in A and B are shown, respectively. Scale bars equal 100 μm in A (applies to A and B), C (applies to C-H). [file 1756-6606-3-36-S5.PDF]
